# Using Machine Learning to Optimize the Quality of Survey Data: Protocol for a Use Case in India

**DOI:** 10.2196/17619

**Published:** 2020-08-05

**Authors:** Neha Shah, Diwakar Mohan, Jean Juste Harisson Bashingwa, Osama Ummer, Arpita Chakraborty, Amnesty E LeFevre

**Affiliations:** 1 Department of International Health Johns Hopkins Bloomberg School of Public Health Baltimore, MD United States; 2 Faculty of Health Sciences Department of Integrative Biomedical Sciences, & Member of the Institute of Infectious Disease and Molecular Medicine University of Cape Town Cape Town South Africa; 3 Oxford Policy Management New Delhi India; 4 Division of Epidemiology and Biostatistics School of Public Health and Family Medicine University of Cape Town Cape Town South Africa

**Keywords:** quality assurance, household survey data, machine learning, monitoring, real-time data, data analytics

## Abstract

**Background:**

Data quality is vital for ensuring the accuracy, reliability, and validity of survey findings. Strategies for ensuring survey data quality have traditionally used quality assurance procedures. Data analytics is an increasingly vital part of survey quality assurance, particularly in light of the increasing use of tablets and other electronic tools, which enable rapid, if not real-time, data access. Routine data analytics are most often concerned with outlier analyses that monitor a series of data quality indicators, including response rates, missing data, and reliability of coefficients for test-retest interviews. Machine learning is emerging as a possible tool for enhancing real-time data monitoring by identifying trends in the data collection, which could compromise quality.

**Objective:**

This study aimed to describe methods for the quality assessment of a household survey using both traditional methods as well as machine learning analytics.

**Methods:**

In the Kilkari impact evaluation’s end-line survey amongst postpartum women (n=5095) in Madhya Pradesh, India, we plan to use both traditional and machine learning–based quality assurance procedures to improve the quality of survey data captured on maternal and child health knowledge, care-seeking, and practices. The quality assurance strategy aims to identify biases and other impediments to data quality and includes seven main components: (1) tool development, (2) enumerator recruitment and training, (3) field coordination, (4) field monitoring, (5) data analytics, (6) feedback loops for decision making, and (7) outcomes assessment. Analyses will include basic descriptive and outlier analyses using machine learning algorithms, which will involve creating features from time-stamps, “don’t know” rates, and skip rates. We will also obtain labeled data from self-filled surveys, and build models using k-folds cross-validation on a training data set using both supervised and unsupervised learning algorithms. Based on these models, results will be fed back to the field through various feedback loops.

**Results:**

Data collection began in late October 2019 and will span through March 2020. We expect to submit quality assurance results by August 2020.

**Conclusions:**

Machine learning is underutilized as a tool to improve survey data quality in low resource settings. Study findings are anticipated to improve the overall quality of Kilkari survey data and, in turn, enhance the robustness of the impact evaluation. More broadly, the proposed quality assurance approach has implications for data capture applications used for special surveys as well as in the routine collection of health information by health workers.

**International Registered Report Identifier (IRRID):**

DERR1-10.2196/17619

## Introduction

Data quality is vital for ensuring the accuracy, reliability, and validity of survey findings. Traditional approaches to monitoring data quality have sought to consider both the *intrinsic* (the data collection tool, implementation and support systems governing its use) and *extrinsic* factors (weather, enumerator-respondent dynamics, community environment) underpinning survey implementation, and in turn, data quality [1]. The quality of survey data starts with the selection of the survey institutions that will support the design and development, sampling, and implementation of the instrument. Decisions on survey tool content, including the selected indicators, language used, and phrasing of both questions and response options, as well as the length of the tool and the broader implementation strategy (tablets versus paper tools, sampling, training, profile, and the number of enumerators, workload), and support structures including supervision and reliability checks, may also influence quality.

Strategies for ensuring survey data quality have traditionally used quality assurance (QA) procedures—defined as “any method or procedure for collecting, processing or analyzing survey data that is aimed at maintaining or enhancing their reliability or validity” [2]. QA procedures usually focus on the intrinsic factors which influence data quality, starting with the sampling (overview of population composition, sampling frame, stratification, size), tool development (reliability of questions, accuracy, understandability of translation), enumerator selection (experience and profile) and training (length, methods, and content of training; enumerator and supervisor evaluation) [2]. These are followed by the piloting and refinement of tools and, ultimately, main survey implementation. QA procedures during implementation concentrate on survey personnel (enumerators, supervisors, coordinators), logistics (travel and team organization), contact procedures (respondent identification/ introduction, consent, refusal rates), enumerator remuneration, supervisor checking procedures, data transfers and checks [2]. Examples of QA procedures during implementation may include reliability checks from supervisors, site visits from senior study personnel, as well as routine data analytics.

Data analytics is an increasingly vital part of survey QA, particularly in light of the increasing use of tablets and other electronic tools which enable rapid, if not real-time, data access. Routine data analytics are most often concerned with outlier analyses that monitor a series of data quality indicators, including response rates, missing data, and reliability of coefficients for test-retest interviews. Dashboards may be used to visualize key tracking indicators and provide a snap-shot of survey implementation status. Collectively, while these procedures help ensure basic data quality, they fall short of optimizing the full potential of rapid data access borne from the use of electronic tools during survey implementation.

Machine learning is emerging as a tool with the potential to enhance real-time data monitoring by identifying trends in data collection that could compromise quality [3]. Machine learning covers a broad array of computationally-intensive methods aimed at detecting patterns in the data, including outliers and subtle trends that would not always be noticed via manual data cleaning and analysis. In the context of QA for survey monitoring, machine learning can be used to classify the data by the severity of outliers using either supervised techniques—with labeled training or pilot data—or a variety of unsupervised techniques [4].

Household surveys are expensive, time-consuming, and resource-intensive. Recognizing this, to date, applications of machine learning as part of household surveys have sought to reduce the expense of surveys by using satellite or phone data to predict socioeconomic distribution in a country [5]. However, little work has been done using machine learning techniques to improve the monitoring of surveys. To address this gap, we aim to outline a comprehensive strategy for monitoring the quality of data emerging from a large population-based household survey in rural India, including the use of machine learning techniques. Study findings are anticipated to shed light on the feasibility and effectiveness of advanced monitoring using machine learning as compared to traditional monitoring techniques.

## Methods

### Survey Description

In late 2018, as part of an impact evaluation of the maternal messaging program Kilkari, a randomly selected sample of 5095 women 4-7 months pregnant, with access to a mobile phone were identified across four districts of Madhya Pradesh, India: Rewa, Rajgarh, Hoshangabad, and Mandsaur [6]. Identified women were administered a structured baseline survey tool that sought to measure their knowledge of reproductive, maternal, newborn, and child health (RMNCH) practices and observe their digital literacy. Following the baseline survey, women were randomized to receive Kilkari messages or not (status quo). In this paper, we focus on the QA procedures for the endline survey administered to women enrolled in the study at 12 months postpartum. The endline survey will be used to capture RMNCH decision making, discussion, knowledge, and practice.

### Study Setting and Population

The study setting in Madhya Pradesh is characterized by disparities in access to education, mobile phones, and health services by gender and geographic location (rural/urban). With a population of over 75 million, Madhya Pradesh is home to over 20% of India’s population. Madhya Pradesh ranks as one of the worst-performing states in India economically (gross domestic product per capita of US $1100 versus US $1709 nationally) and in terms of health outcomes, particularly concerning child nutrition. In 2015, only 35% of children were breastfed within one hour of birth, and 58% of children were exclusively breastfed until 6 months [7]. While over half of pregnant women attended antenatal care (ANC) in the first trimester, only 36% received the recommended four ANC visits [7]. Health behaviors and care-seeking practices differ markedly between urban/rural areas and are underpinned by high rates of illiteracy (41% of women, 18% of men) and poor access to mobile phones among women [7]. In 2015, 19% of rural and 50% of urban women reported having access to a mobile phone [7].

Our trial population consists of women who have given birth in the past 1 year after being enrolled while they were  12-34 weeks of gestation, and all participants are at least 18 years of age, speak and understand Hindi, and own or have access to a mobile phone during the morning or afternoon. Our sample ranges from the ages of 18-44 years, with 61% of the sample being between the ages of 20-25. In terms of socioeconomic profile, 21% of our sample is “general class,” which is socially privileged, 47% are “other backward class,” which is somewhat marginalized, and the remainder are from highly marginalized groups—scheduled caste (20% of the sample) and scheduled tribe (11% of the sample). Hindu individuals comprised 95% of the trial sample, and 11% had not received any formal education, 6.5% had 1 to 4 years of schooling, 17.8% had between 5 and 7 years, 56.0% had between 8 and 12 years, and 8.7% had more than 12 years.

### Overview of Quality Assurance Procedures

**Figure 1:**
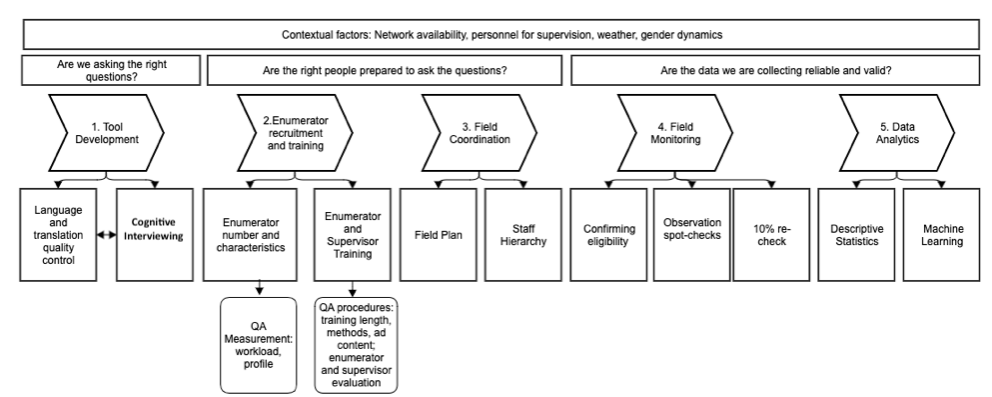
Conceptual Framework of methods to improve data quality from Kilkari womens’ household survey.

The household survey monitoring strategy aims to identify biases and other impediments to data quality and includes seven main components: (1) tool development, (2) enumerator recruitment and training, (3) field coordination, (4) field monitoring, (5) data analytics, (6) data feedback loops for decision making, and (7) outcome assessment. In the framework below, we have outlined the processes we aim to complete in an effort to ensure survey data quality (Figure 1).

#### Tool Development

Tool development assumed six essential steps: (1) linking intervention content with key indicators across behavior change outcomes for decision making, discussion, knowledge and practice, (2) drawing survey content from standardized and/or validated survey tools, literature, and/or expert review, (3) translation, (4) cognitive interviewing, (5) pilot testing, and (6) computer-assisted personal interviewing (CAPI) checking.

Our tool development began with looking at our key indicators on knowledge, practice, discussion, and decision making around topics relevant to pregnant and postpartum women such as family planning, infant and young child feeding, and newborn care. We then chose to assess these indicators using selected questions from previously completed standardized surveys. These questions were reorganized, reworded, and translated. The survey was a close-ended quantitative survey with single-answer multiple-choice questions as well as multiple-answer multiple-choice questions. Questions for which other options could be provided had an “other specify” text box. We chose to go beyond the traditional pilot testing methods and also use cognitive interviews to test our tools. Cognitive interviewing involves first asking the survey question as it is written, recording the respondent’s answer, then using verbal probes to determine if the respondent is understanding the question and providing an answer in the manner intended by the researchers [8]. We used this method to assess if our tool’s major sections on knowledge and behavior of family planning and infant and young child feeding were accessing the cognitive domains we intended them to [9]. Through this work, we revised our questions to improve their comprehensibility for our sample population (women in rural Madhya Pradesh).

During training, we spent time reviewing the paper versions of the tool as well as ensuring that our CAPI tablet versions, programmed using CSPro software, mirrored them well and adhered to the skip logic we had outlined. Both paper and tablet versions of the survey took on average about 1.5 hours to complete. The tablet version of the survey had just one question at a time appear on the screen. The process of checking the tablet version of the tool continued before and after piloting these versions in the field to ensure we had made all necessary changes in both language and logic before data collection with our end-line sample began. All survey data will be collected on tablets.

As part of the tablet-based data collection forms, we added a few parameters that were not part of the paper tools. These included time-stamps for the start and end of modules and time flags at the beginning and end of questions involving in-depth probing. GPS fields were added to the end of the tablet-based tool to track location.

#### Enumerator Recruitment and Training

Enumerator recruitment depends on survey size and timeline, as well as the characteristics of the enumerator. The experience and characteristics, including age, gender, caste, ethnicity, education, and geographic origins of an enumerator can influence not only their understanding of survey questions but also the implementation of the survey, including how they explain questions to and interact with respondents. Survey enumerators will be women fluent in Hindi, between 21-35 years of age, with education levels ranging from current college students to current PhD students, and 0-12 years of survey experience. To monitor the effects of enumerator characteristics and experience on data quality, we will start by administering a short survey during training to formulate their profile, and then as survey data are collected, use these profile data to understand potential associations between profile data and survey data quality.

To ensure that adequate numbers of enumerators are recruited, we have projected the number of interviews that need to be completed weekly by geographic area and considered the time it takes to administer each survey coupled with the time required to travel to and locate respondents. Additional factors such as enumerator attrition, along with the need to conduct repeat visits to locate respondents or complete partially completed surveys, will be factored in.

Once an adequate number of enumerators and supervisors are recruited, training will be key to optimizing survey implementation and ensuring data quality. Training will be led by the in-country field manager, a physician with >30 years of survey management experience. Additional support will be provided by study team members who participated in the development of the tool in its early stages. The training spanned 14 days and involved classroom-based lectures, quizzes, role-playing exercises, and field-based piloting. The tool covered multiple modules, including family planning methods, infant and young child feeding, and the immunization schedule. First, the didactic lectures covered the information on the domains covered in the tool. Then the field manager, along with support from various members of the study team who were instrumental in developing the tool, walked through the tool, including the questions, responses, and how to administer the question in a detailed manner. The session leader verified the data collectors’ understanding and asked them to clarify any lingering questions they may have had. After explaining and discussing the questions with the group, the enumerators conducted mock-interviews amongst themselves. A few quizzes on these topics were administered to ensure that the enumerators and supervisors were familiar with these health areas and associated vocabulary. In order to provide more real-world practice, there were two field pilot days in nearby villages that resembled the study population but were outside the study population, one with paper forms and another with the tablet version of the surveys with which the enumerators could practice. These pilot test days allowed the enumerators to become comfortable using the tool with live respondents, as well as allowing them an opportunity to comment on any issue in the tool, including language and flow.

#### Field Coordination

Beyond the development of the tool and enumerator selection and training, the quality of data is improved by strong field coordination. Field coordination is built on two main components: field and logistics planning as well as coordinating the monitoring of data collection through supervisors and checks. First, key eligibility criteria for interviews need to be kept in mind when planning field logistics. Women will be interviewed if they have completed 12 months postpartum or longer; thus, field planning will require that enumerators be spread across four districts of Madhya Pradesh to capture women as they reach their expected date of eligibility. Confirming women’s eligibility will require reliability monitoring, as outlined below. Once an eligible woman is identified, supervisors are given her identification and location information so they can coordinate their team of enumerators to her location and manage any follow-up visits as necessary depending on her availability.

**Figure 2:**
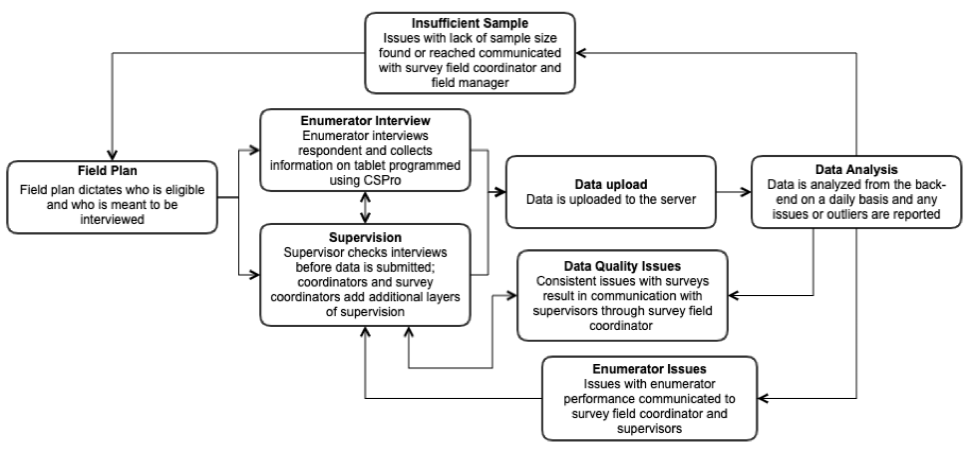
Data flow and feedback in Kilkari women’s survey.

A clear hierarchy in quality assurance activities as part of field operations is key for ensuring the collection of high-quality data. The field team will include 35 enumerators, 9 supervisors, and 2 coordinators, a survey coordinator, and a field manager. The male supervisors will be paired with a team of enumerators, consisting of 3-4 female enumerators, and will be in charge of checking the data as it is collected. The coordinators track adverse events and logistics and will conduct spot checks. The survey coordinator will monitor the data and relay any issues back to the larger research team. The field manager will handle training as well as ensure the field plan is in place. With clear roles in place, the field manager and survey coordinator will be best able to plan the fieldwork and monitoring, respectively. The data flow and feedback processes are outlined below (Figure 2).

#### Field Monitoring

Field monitoring consists of both confirming the eligibility of respondents as well as spot checks. Both these aspects happen at each of the five levels of field hierarchy: (1) enumerator, (2) immediate field supervisor, (3) coordinator, (4) field-level data quality supervisor, and (5) back-end data quality supervisor. At the enumerator level, any issues or red flags identified by enumerators will be reported back to their immediate supervisor who assigned them to the interview; these could include issues of eligibility or issues with tablets or other interview processes. Immediate field supervisors will focus principally on locating respondents and monitoring interviews. The former occurs based on the respondents the field coordinators give each supervisor—the respondent is located, and eligibility is confirmed. Then the supervisor may monitor part or all of the interview either through spot-check observation or form check and keep track of completion or any needed follow-up visits. The coordinators will manage larger village/block level logistics to ensure all eligible participants in an area have at least been visited once on a trip and also complete random spot-checks as their logistics duties allow. The field level quality supervisor will complete spot-checks as well as follows-up on any systemic issues found in both eligibility checking or back-end data monitoring either by individually speaking with enumerators/supervisors or discussing any data quality issues during debriefing meetings with the team. Finally, the back-end data quality supervisor will check weekly data uploads for any entry errors relating to eligibility, such as incompatible birth age or incorrect unique identifier. They will also assess mechanistic indicators such as time to interview completion or rate of skips or “don’t know” responses that warrant further observation of participating enumerators. The larger research team will conduct monthly checks on the field teams.

Beyond eligibility confirmations and observation spot-checks, our second stage of reliability monitoring will involve a 10% resample to ensure enumerators are asking the questions correctly so that we are getting consistent answers. A set of 15-25 questions will be randomly selected from a bank of 30, and the 10% sample will also be randomly selected from the total eligible population that has completed interviews. The recheck will be completed by a different enumerator either later in the day or a day later after the original interview has taken place.

#### Data Analytics

Data analytics will focus on descriptive and outlier analyses.

Descriptive analyses will focus on the conduct of basic frequencies across all questions in the survey to ensure that questions are not being unexpectedly skipped. Beyond checking the frequencies across the variables, we will also examine any anomalies, such as incorrect unique identifiers or ineligible respondents based on date, every week. These frequencies will be examined after each district in the survey is halfway complete.

The outlier analysis aims to identify anomalies in the data, which could indicate gaps in quality. Outlier analysis will start with the selection of features, and depending on these features and their distributions (parametric/non-parametric), several techniques will be explored for outlier detection including numerical outliers, Z-score, linear models, probabilistic and statistical models, as well as unsupervised machine learning (k-means clustering). Analyses will draw from the following data sources: enumerator completed survey tools from piloting, enumerator completed survey tools from the main field implementation, enumerator profile survey, and the recheck survey. The following steps will be undertaken:

##### 1. Selecting Features Necessary to Examine Gaps in Quality

Data features will be identified from the enumerator collected survey tools during implementation. They may include (a) time to complete the overall questionnaire, select modules, and individuals questions, (b) frequency of enumerator selection of the response option ‘don’t know,’ and (c) skip patterns. While time-stamping all individual questions in the data collection tools will not be possible, select priority questions (eg, dietary recall) will be time-stamped, along with the start-stop times by module and for the overall time to complete the tool in its entirety. Data from enumerator collected survey tools during implementation will be linked with data on the enumerator profile and descriptive statistics used to identify outliers and as needed feedback data requiring follow-up. Each of these features and the indicator proposed to measure each is summarized in Table 1. Machine learning algorithms will ultimately be developed through the additional steps described below.

##### 2. Obtaining Labeled Data

Labeled data are essential in supervised machine learning. Labeled data can assume a variety of forms; in this case, we have data from enumerators completed on their own without a respondent as fast as they possibly can. These data are then labeled as ‘false’ data to be used in our supervised learning algorithms. By comparison, another labeled dataset will come from data captured during the pilot testing of tools. An alternative strategy for obtaining labeled data through data augmentation will also be explored. Data augmentation is the process of supplementing a dataset with similar data that is created from the information in that dataset.

##### 3. Building Models

This data will be split randomly into a training set and a testing set. As predictors of the models, we will use the features listed in step 1, and the data will be labeled as “false” or “true.”

Two steps will be undertaken to build models: (1) develop the best models for each learning algorithm using the training dataset and cross-validation methods and (2) apply the best performing algorithm on the test dataset for an unbiased evaluation. During the learning (training) process, k-fold cross-validation methods will be used to avoid overfitting and assess model performance. The k-fold cross-validation method involves splitting the dataset into k-subsets, which are, in turn, held out while the model is trained on all other subsets. The process is complete when accuracy has been determined for each instance in the data set, and an overall estimate of accuracy has been generated. Once the learning step is accomplished on the “training” set, we have trained models. Each model will be tested using the originally withheld testing set.

The algorithms under consideration are listed below in Table 2. We aim to train using all methods to identify the best algorithm for our data. Methods to assess the models, compare their attributes, and formulas to compare the results of the learning algorithms are similar to those presented in a study by Mohan et al [3]. That study also describes comparisons of the strengths of these various algorithms [3].

**Table 1:** Biases in survey completion that can be captured using machine learning.

Biases	Indicator	Numerator	Denominator
Rushed completion	Percent of time to complete survey, sections, and individual question as compared to time recorded during piloting	Time measured during the pilot—actual time in seconds	Pilot time
CAPI^a^ entry error/ Fabricated responses	Percent difference from 10% resample	Questions that are different in 10% resample	Total number of questions resampled
Respondent bias	Mean time taken to complete questions among respondents in differing sociodemographic characteristics	N/A^b^	N/A
Skipping of sections	Percent of skips across all potential skip options throughout the tool	Opportunities where a skip was implemented	Total opportunities where a question or section can be skipped
Misunderstanding of content	Percent of “don’t knows” across all questions and by section per respondent or enumerator	Questions with “don’t know” answers	Total number of questions with “don’t know” options

^a^CAPI: computer-assisted personal interviewing.

^b^N/A: not applicable.

**Table 2:** Machine learning algorithms.

Algorithm		Description	Intended application
**Supervised**			
	Logistic regression	Classification (nonlinear model)	Classification of times, “don’t knows,” and skips used by enumerator characteristics and respondent characteristics
	Linear discriminant analysis	Classification (linear model). It is a linearization of Gaussian naïve Bayes.	Classification of times, “don’t knows,” and skips used by enumerator characteristics and respondent characteristics
	Support vector machines	Support vector machines are techniques based on the calculation of the maximum margin hyperplane for classification problems.	Classification of times, “don’t knows,” and skips used by enumerator characteristics and respondent characteristics
	Classification and regression trees	A predictive model that consists of leaves that represent the target and branches that represent conjunctions of input features. Considered a subset of decision trees. Random forests operate by constructing multiple decision trees during training and aggregating their results to avoid overfitting by single trees.	Classification of times, “don’t knows,” and skips used by enumerator characteristics and respondent characteristics
	Naïve Bayes	Classification model based on probabilities.	Classification of times, “don’t knows,” and skips used by enumerator characteristics and respondent characteristics
	Neural networks	Neural networks are powerful models for machine learning. They are a generalization of linear and nonlinear models	Classification of times, “don’t knows,” and skips used by enumerator characteristics and respondent characteristics
**Unsupervised**			
	K-means	K-means clustering is a way to use data to uncover natural groupings within a heterogeneous population	Grouping of Enumerators’ times, “don’t knows,” and skips used by enumerator characteristics and respondent characteristics

#### Feedback Loops for Decision Making

**Figure 3:**
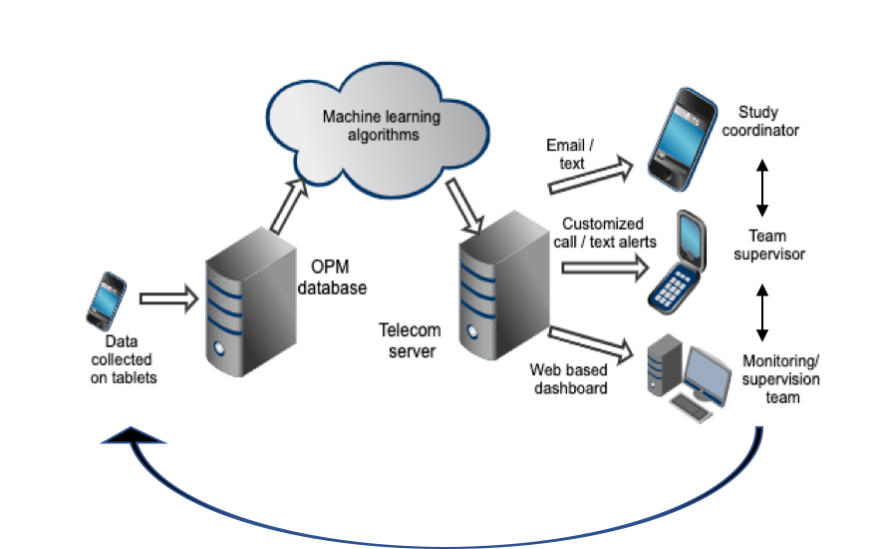
Automated feedback loops.

Data quality issues identified during analysis will be fed back to the survey coordinator and survey team in the field. The immediate strategy for feedback will require that the data quality supervisor identifies the problem and communicates with the study coordinator via phone and email; the coordinator will then speak with the team supervisor and the enumerator (Figure 2). The study team is also exploring the possibility of automated feedback loops to capture data quality gaps identified by the machine learning algorithms and send QA issues via email or text to the study coordinators, customized calls or text alerts to team supervisors, and provide the monitoring and supervision teams with access to a web-based dashboard for data visualization (Figure 3).

Depending on the magnitude of the issue identified and its frequency, an appropriate response will be devised and may include trouble-shooting issues with CAPI/tablets, enumerator retraining or encouragement as needed, or in serious circumstances, re-interviewing the respondent. A more pervasive issue may require a team meeting where a problem with a certain question or set of the respondent population needs to be dealt with differently. Depending on the severity of the issue, the survey coordinator will be in charge of deciding how best to handle it and recheck the data to ensure the problem has been resolved.

#### Outcome Assessment

Given that this is a small pilot study of this SMS feedback system, we will be assessing acceptability and feasibility. To examine if the SMS system reduces the amount of time to address errors, we will look at the difference between the date the error was made and the date it was resolved. Additionally, we plan on conducting qualitative interviews with the coordinators and supervisors to understand their perceptions of the SMS format of feeding information back and soliciting data on feasibility and acceptability. We hope to conduct in-depth interviews and focus group discussions with field staff towards the end of the data collection period to learn this information from them after they have been using the SMS system for an extended period.

## Results

This study has been approved by the Johns Hopkins Bloomberg School of Public Health Institutional Review Board and the India-based Sigma Research and Consulting review.

## Discussion

Machine learning has the potential to enhance routine QA methods for surveys, as well as the routine capture of health information through data capture applications. The proposed QA strategy for the Kilkari Impact Evaluation aims to comprehensively improve data collection quality through innovations in the tool development stage, such as cognitive interviewing, as well as in the monitoring stage through the use of machine learning. By complementing traditional monitoring back-end checks, machine learning has the potential to enhance survey monitoring by identifying biases in survey data collection, which may hamper data quality and, in turn, emerging findings. The potential use of automated feedback loops via text and email may serve to enhance the timeliness of feedback at the point of data collection and, in turn, improve efficiencies in data collection by minimizing the need to return to geographic areas after teams have left. The QA approach outlined in this protocol may have additional implications for study teams comprised of researchers spread across wide geographies, enhancing remote QA and enabling greater confidence in emerging data.

The limitations of this approach include the parameters used for the machine learning approach—ideally more time-stamps and information on tapping forward or back off a screen would provide more detail on the timing and mechanics of each survey completed. In some cases, an interview may be interrupted by factors outside the enumerator’s and respondent’s control such as a baby crying or a neighbor calling; however, the enumerators are encouraged to do their best to complete the survey in one sitting and include comments on hindering circumstances that extend the length of the interview at the end of the survey. Additionally, some skips in the surveys and “don’t know” responses are necessary and unavoidable; thus, these measures could be ambiguous at times. Given the nature of our multi-select tool, we expected enumerators to be probing for answers and using “other specify” for ambiguous answers and using the “don’t know” option minimally. However, this parameter is specific to our survey, and it could be that in another type of tool, other parameters are used to indicate rushed or low-quality interviews. This difference is true of other steps as well, such as which time-stamps to collect, what kind of labeled data to obtain, and the types of responses that are useful for quality control. Quality assurance needs to be tailored for each survey.

In large surveys, such as the Demographic and Health Surveys (DHS) and Multiple Indicator Cluster Surveys (MICS), traditional monitoring techniques are used. DHS quality checks during fieldwork focus on assessing quality based on the number of questions answered, recoding, and eligible sample met. Additional measures such as age displacement, response rates, and completeness of data are relayed back to the data collectors to improve quality during the period of data collection [10]. After data entry, the number of questionnaires is checked against the number expected according to the sample design, and double entry is conducted to minimize entry mistakes. The entered data are checked for further issues, and variables determined to be missing at random are calculated using needed imputations. MICS follows similarly strict guidelines with interview teams headed by supervisors and accompanied by measurers who are in charge of taking measures of weight and water quality. Data quality checks focus on the number of respondents, deviations from the average weight of children interviewed, ages of respondents, and nonresponse rates [11]. While these monitoring approaches are widely used and time-tested, machine learning could further strengthen the monitoring approaches used in these large surveys.

Thus far, the use of machine learning in household surveys in Lower-middle-income country settings is limited. One set of researchers has used tree-based machine learning methods to model and predict nonresponse in surveys [12]. Similarly, a working paper from the United Nations Economic Commission for Europe examined the use of machine learning methods to develop data editing and imputation [13]. Both these studies focus on the use of machine learning to counteract and improve household surveys with sparse data. Another study with objectives similar to ours sought to measure the rate of enumerators falsifying information in Tanzania using machine learning [14]. Our study is an extension of the work done in Tanzania by automating the process of quality assurance to send survey errors back to the field team.

Machine learning holds great value for other aspects of international development beyond survey work as well. In 2016, Goldblatt et al used satellite imagery in India to examine areas of urbanization in a rapidly developing country [15]. A World Bank Group additionally reported using natural language processing to study differences between genders when deliberating topics in village meetings transcripts across Tamil Nadu [16]. Given the expanding use of these methods in international development and survey work, it is only fitting that we take them a step further to assist with monitoring incoming data.

### Conclusions

The comprehensive QA strategy outlined in this protocol aims builds on traditional approaches to survey QA through the use of machine learning methods to improve data monitoring, and in turn, quality. The approach undertaken is anticipated to improve the rigor of impact evaluation data currently being collected in four districts of Madhya Pradesh, India, as part of the Kilkari Impact Evaluation. Broader learnings are anticipated as this protocol is additionally envisaged as a use case for the application of similar methods to improve the quality of data emanating from data capture digital health solutions currently being used by health workers throughout India.

## Abbreviations

ANC: antenatal care
CAPI: computer-assisted personal interviewing
DHS: Demographic and Health Surveys
QA: quality assurance
RMNCH: reproductive, maternal, newborn and child health
MICS: Multiple Indicator Cluster Surveys
